# Ultralong well-aligned TiO_2_:Ln^3+^ (Ln = Eu, Sm, or Er) fibres prepared by modified electrospinning and their temperature-dependent luminescence

**DOI:** 10.1038/srep44099

**Published:** 2017-03-07

**Authors:** Hongquan Yu, Yue Li, Yang Song, Yanbo Wu, Xijie Lan, Shimin Liu, Yanning Tang, Shasha Xu, Baojiu Chen

**Affiliations:** 1 College of Environmental and Chemical Engineering, Dalian Jiaotong University, Dalian, Liaoning 116028, P. R. China; 2 Department of Physics, Dalian Maritime University, Dalian, Liaoning 116026, P. R. China

## Abstract

Electrospinning has emerged as an attractive technique for the fabrication of ultrafine fibres in micro-/nano-scale fineness: however, it remains a significant technological challenge to assemble aligned fibre arrays via an conventional electrospinning method due to the inherent whipping instability of the polymeric jet. We herein have first developed a simple modified electrospinning method with which to prepare ultralong (>300 mm) well-aligned inorganic fibre arrays, *i.e*., using an ultrahigh molecular weight polymer to suppress or eliminate the whipping motion of the electrospun jet, has emerged as a facile approach for the continuous fabrication of well-aligned, ultralong fibres through simply using a rotating cylinder as the collector (it was not found necessary to use a very high rotating speed, extra magnetic, electrical field) in the electrospinning process. As result, the ultralong well-aligned TiO_2_:Ln^3+^ (Ln = Eu, Sm, or Er) fibre arrays can be obtained from ultrahigh molecular weight poly(ethylene oxide), tetra-n-butyl titanate (Ti(OC_4_H_9_)_4_) and lanthanide nitrate in the modified electrospinning approach. The grow mechanism and luminescent properties of these ultralong well-aligned TiO_2_:Ln^3+^ fibre arrays were also investigated.

In past twenty years, one-dimension (1-d) titania nanostructures have attracted rapidly increasing attention due to their important role in applications related to gas sensing, optoelectronic devices, biomedicine, in the fabrication of solar cells and batteries^1^,^2^,^3^,^4^,^5^,^6^,^7^,^8^,^9^. However, disordered structures are problematic for use in device fabrication in such areas as microelectronics, photonics. Thefore, researchers has explored novel methods including the hydrothermal route, the AAO template method, the electrochemical approach etc. to improve the alignment of 1-d titania nanostructures in past decades^10^,^11^,^12^. However, the yield production of the 1-d titanium arrays obtained from these methods is much less, not in sufficient quantities to satisfy commercial demand. And thses titanium arrays in length have just several hundred microns. It is kown that, the electrospinning technique is a effective way to produce polymer or inorganic fibres with diameters as small as 10 nm and up to a few microns in diameter. This approach is inexpensive, easily operated, non-polluting, and available suits for industrial production. The length of the nanofibres depends on the time of electrospinning and can be thousands of kilometres (in theory)^13^. However, under normal conditions, bending instabilities of polymer jet are was the primary cause of generate randomly oriented fibres with various diameters and structures during the electrospinning process^14^,^15^. In past decades, researchers has explored novel methods including mechanical, electrostatic, and other ways of improving the alignment of electrospun nanofibres^16^,^17^,^18^. These methods comprising largely of using high speed collectors and/or through manipulating the electrical field have been devised and shown to work well in preparing aligned electrospun fibres and patterns. However, the set-up used is somewhat complicated or the alignment of as-spun fibres cannot be maintained with increasing collection time due to the varying electrical conductivity of the collector caused by increased numbers of fibre layers. Therefore, it remains a significant technological challenge to assemble aligned fibre arrays via an conventional electrospinning method.

Recent years, the nanoscale lanthanide doped compounds in particular 1-d nanostructures have attracted much research interest for their broad potential applications-e.g. color display, solar cells, ultrasensitive detection and *in vivo* imaging^19^,^20^,^21^,^22^,^23^. Thus, developing an strategy to easily obtain mass-scale manufacturing of the 1-d lanthanide doped nanostructures is very significant to satisfy practical applications. In the present work, we develope a improved the conventional electrospinning technique to large-scale easily produce ultralong parallel well-aligned TiO_2_:Ln^3+^ (Ln = Sm, Eu, or Er) fibre arrays. In this method, the ultrahigh molecular weight PEO was exploited to eliminate the occurrence bending instability of the electrospinning jet to achieving highly aligned fibre assemblies. In addition, the grow mechanism and luminescence properties of well-aligned TiO_2_:Ln^3+^ fibre arrays were also investigated. This simple approach can achieve ultra-long aligned inorganic nanofibres and nanostructures arrays in a straightforward and scalable fashion, suitable for a variety of practical applications.

## Experimental Procedure

Some 2.0 g of tetra-n-butyl titanate (Ti(OC_4_H_9_)_4_) was dropped in the mixed solvent with 5 ml of ethanol and 15 ml of CH_2_Cl_2_ under magnetic stirring for 20 min. Then a certain amount of rare earth nitrate such as Eu(NO_3_)_3_, Sm(NO_3_)_3_ · 6H_2_O, or Er(NO_3_)_3_ · 6H_2_O was added to this mixture, respectively. The molar ratio of Eu^3+^ to Ti^4+^ was 1.0, 3.0, 5.0, and 10.0 mol %, respectively. The molar ratio of Sm^3+^ to Ti^4+^ was 1.0, 5.0 and 10.0 mol%, respectively. The molar ratio of Er^3+^ to Ti^4+^ was 1.0 mol%. After 20 min, an amount of PEO was added to the aforementioned mix solution, followed by magnetic stirring for about 1 h to obtain the final electrospinning solution. Supplementary Figure 1 shows the schematic diagram of the electrospinning set-up, which consisted of three major parts: a high-voltage power supply, a spinneret (plastic needle), and a collector (rotating plastic drum). The precursors of TiO_2_:Ln^3+^ Ln = Sm, Eu, or Er) fibre arrays were obtained by electrospinning with a distance of 200 mm between the spinneret tip and the collector, an applied voltage of 5.0 to 8.0 kV at a rate of rotation of the drum of between 500 to 1400 rpm (1 rpm was equivalent to a linear speed of 0.0067 m/s). The as-prepared precursor fibres of the TiO_2_:Ln^3+^ array were taken off and calcined at a heating rate of 10 °C · h^−1^ in air to remove organic components, thus forming ceramic TiO_2_ Ln^3+^ fibres.

The crystal structure of the samples was studied by powder X-ray diffractometer (XRD, Shimadzu, XRD-6000) with Cu Kα radiation (*λ* = 0.15406 nm). In the X-ray diffraction measurements, the scanning region was 20° ≤ 2 *θ* ≤ 80°, and the scanning step size was 0.02°. The morphology of the samples was observed by field emission scanning electron microscope (SEM, SUPRA 55). The TEM images, HR-TEM images, and selected area electron diffraction patterns (SAED) of the HR-TEM images were recorded on a JSM-2010 transmission electron microscope (JEM, Japan) under a working voltage of 200 kV. The excitation and emission spectra were recorded on a Hitachi F-4600 spectrophotometer equipped with a continuous 150 W Xe arc lamp: for comparison between different samples, the emission spectra were measured at a fixed pass of 0.2 nm with the same instrument parameters (a 2.5 nm excitation split, a 2.5 nm emission split, and a 400 V photomultipier tube voltage).

## Results and Discussion

Changing the electrospinning conditions, such as the applied voltage, the PEO content, and the rate of rotation of the collecting drum, all exerted a significant influence on the size and morphology of the precursor fibres of TiO_2_. Supplementary Figures 2 to 5 show the FE-SEM images of the TiO_2_ precursors fibres prepared under different conditions Accordingly, it can be found that the obtained TiO_2_ precursor fibres have random structures with regards their spatial orientation when the rate of rotation of the collecting drum was less than the 800 rpm, while aligned TiO_2_ fibres were obtained when the rate of rotation of the drum exceeded 1100 rpm. The orientation of the as-spun fibres was improved with increasing rate of rotation of the collecting drum. Besides, Supplementary Fig. 2(e) and (f) further confirm that the precursor TiO_2_ fibres are well-aligned fibre arrays. Within the range studied, an increased PEO and Ti(OC_4_H_9_)_4_ concentration caused the diameter of the precursor fibres to increase gradually. The PEO concentration was 1.0, 2.0, 2.5, and 3.0 wt%, the corresponding average diameters of the TiO_2_ precursors fibres were 3.0 μm, 4.2 μm, 5.0 μm, and 6.0 μm, while the Ti(OC_4_H_9_)_4_ concentration was 8.0, 10.0, or 20.0 wt%, the corresponding average diameters of the TiO_2_ precursors fibres are 3.5 μm, 5.0 μm, and 15.0 μm, respectively. Varying the applied voltage exerted no obvious influence on the diameter of fibres, but the orientation of the as-prepared fibres proved to be dependent on the electrospinning voltage. When the applied voltage was too small (<5 kV), the spinning of the jet from the spinneret became more difficult due to the viscoelasticity of the electrospun solution: as the applied voltage increased (>10 kV), the orientation of fibres became increasingly random due to bending instability of the electrospun jet. Most importantly, it was observed that, when a little rare earth nitrate (such that the molar ratio of RE^3+^ to Ti^4+^ was less than 10.0 mol%) was added to the above electrospun solutions, which barely caused any variation in the morphology of the precursor fibres of TiO_2_ under the same experimental conditions.

To obtain well-aligned fibre arrays of pure TiO_2_:Ln^3+^, we submit the as-prepared aligned precursor electrospun fibres to an annealing treatment. After being annealed, the PEO content was removed from the precursor fibres and inorganic TiO_2_:Ln^3+^ fibre arrays were formed. Supplementary Fig. 6 shows the XRD patterns of the aligned TiO_2_:Ln^3+^ fibre array after annealing. It can be seen that the anatase phase of TiO_2_ was formed after calcining at between 500 and 600 °C. The diffraction peaks at 2*θ* values of 25.3°, 37.8°, 48.1°, 54°, 62.8°, 68.8°, and 75.1° belong to the diffraction of the (101), (004), (200), (105), (204), (116), and (215) crystal faces of anatase TiO_2_ (JCPDS card no. 89–4921). With increased calcining temperature, the phase transformation from anatase into rutile TiO_2_ occurred. The diffraction peaks at 2*θ* values of 27.5°, 35.7°, 41.1°, 45°, 54.7°, 57.8°, 65.4°, and 69.8° came from diffraction of the (110), (101), (111), (210), (211), (220), (310), and (301) crystal faces of rutile TiO_2_ (JCPDS card no. 89–4920). When the calcining temperature was greater than 800 °C, the diffraction peaks of anatase TiO_2_ disappeared and all diffraction peaks were assigned to the rutile TiO_2_. In all XRD patterns, no additional Eu^3+^, Sm^3+^, or and Er^3+^ diffraction peaks were observed. These results indicate that the RE ions are embedded within the TiO_2_ lattice.

**Figure 1:**
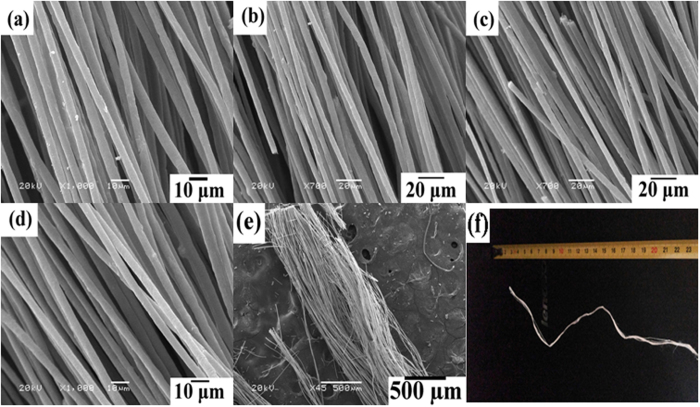
(**a**,**b**,**c** and **d**) are SEM images of well-aligned TiO_2_:Eu fibre arrays from the 1.0 mol%, 3.0 mol %, 5.0 mol%, and 10.0 mol % of the Eu^3+^ concentration, respectively. (**e**) and (**f**) show an SEM image and photograph of large-scale well-aligned TiO_2_:1.0 mol% Eu fibre arrays, respectively. The concentration of PEO of was fixed at 2.5 wt %. The applied voltage was 8.0 kV and the collection distance was 20 mm. The rate of rotation of the collection drum was 1400 rpm. 1 rpm was equivalent to a linear speed of 0.0067 m/s. The as-prepared well-aligned fibres were taken off and calcined at a heating rate of 10 °C · h^−1^ and kept at 500 °C in air for 4 h.

**Figure 2:**
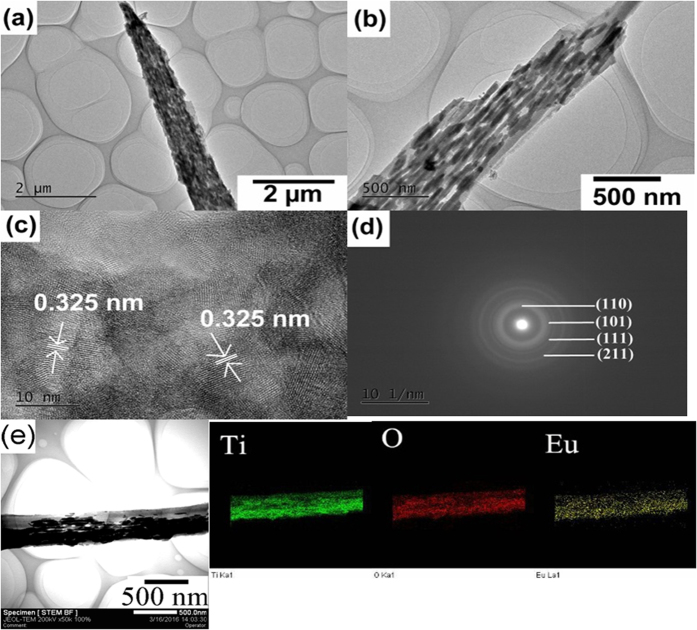
(**a**,**b** and **c**) are lower magnification, image of the end, and HR-TEM images of a single pure TiO_2_:Eu fibre; (**d**) is an SAED pattern of the single pure TiO_2_:Eu 1.0 mol % Eu fibre. (**e**) STEM images and EDX elemental mapping of Ti, O and Eu of TiO_2_:Eu fibre. The as-prepared well-aligned fibres were taken off and calcined at a heating rate of 10 °C · h^−1^ and kept at 800 °C in air for 4 h.

Figure 1(a) to (f) show SEM images of well-aligned pure TiO_2_:Eu^3+^ fibre arrays obtained by calcining at 500 °C. From these images, it can be seen that the morphologies of the ceramic nanofibres were uniform. The average diameters of the single fibree were estimated to be 1.5–2.0 μm for all samples. With increasing rare earth ion dopant concentration, their average diameters were almost unchanged. The large-scale SEM image and photograph further confirm the fact that the TiO_2_:Eu^3+^ fibres exhibit a well-aligned orientation and were ultralong. After annealing at 800 °C, these TiO_2_:Eu^3+^ fibres remained continuous and aligned. The TEM image of a single TiO_2_:1 mol% Eu^3+^ fibre displayed in Fig. 2: the diameter of fibre is about 1.5 μm, and it is composed of well-aligned TiO_2_:Eu nanowires of approximately 50 nm in diameter. Figure 2(c) shows the HR-TEM image of a single rutile TiO_2_:Eu nanowire from the fibre. The crystal lattice fringe with a spacing *d* of approximately 325 nm can be observed directly, which corresponds to the (110) crystal face of rutile TiO_2_, which is consistent with the XRD data. The selected area electron diffraction pattern (SEAD) of the corresponding microbelt is illustrated in Fig. 2(d) which shows the polycrystalline rings which can be indexed against rutile TiO_2_. Figure 2(e) shows the scanning TEM (STEM) and corresponding energy dispersive X-ray spectroscopy (EDX) elemental mapping images, which confirmed that Ti, O, and Eu were distributed on the TiO_2_ fibre surface, consistent with the EDX spectrum (Supplementary Fig. 7). A very similar morphology and size were also observed from pure TiO_2_:Sm^3+^ and TiO_2_:Er^3+^ fibre arrays (Supplementary Figs 8 and 9). The morphology and size of the single fibre were without significant changes with increasing Ln^3+^ dopant concentrations (less than 10% molar concentrations).

It is known that, for such an electrospinning solution, the molecular weight of the polymer has a significant effect on the rheological and electrical properties, such as viscosity, surface tension, and conductivity. This is one of the most important solution parameters affecting the morphology of an electrospun fibre. Therefore, it is very difficult to prepare aligned fibres from low molecular weight PVA, PVP, PMMA, PS, and PEO when working without a high-speed rotating drum (approximately 2000 rpm), auxiliary conductors, and an external magnetic or electric field^24^,^25^,^26^,^27^,^28^. In previous reports, the PVP was used as a structure-directing template and would result in a random orientation of electrospun TiO_2_:Ln^3+^ fibres and the single fibres consisted of the tight connection of multiple irregular TiO_2_:Ln^3+^ nanoparticles^29^. In the present work, we exploited the viscoelasticity contributed by chain entanglement of the ultrahigh molecular weight PEO to eliminate the occurrence of bending instability of the electrospinning jet to achieving highly aligned fibre assemblies. And the PEO as the structure-directing template was used to obtain TiO_2_:Ln^3+^ nanorods arrays in single TiO_2_:Ln^3+^ fibres. A possible mechanism for the formation of well-aligned TiO_2_:Ln^3+^ fibre arrays, is shown in Supplementary Fig. 8. First, the amorphous TiO_2_ sheaths formed at the surface of precursor fibres due to hydrolysis of Ti(OC_4_H_9_)_4_ in air during the electrospinning process (Supplementary Fig. 8(a)). Then, as-spun precursor fibres were annealed. Many ultrafine crystallites were combined into small nanocrystallites through mass transport and the PEO tended to be solidified with increasing temperature (Supplementary Fig. 8(b)). Finally, many small nanocrystallites were further connected to generate TiO_2_ nanorods due to the restriction of PEO rather than through contact points among these small different nanocrystallites to form a randomly oriented structure (Supplementary Fig. 8(c)).

**Figure 3:**
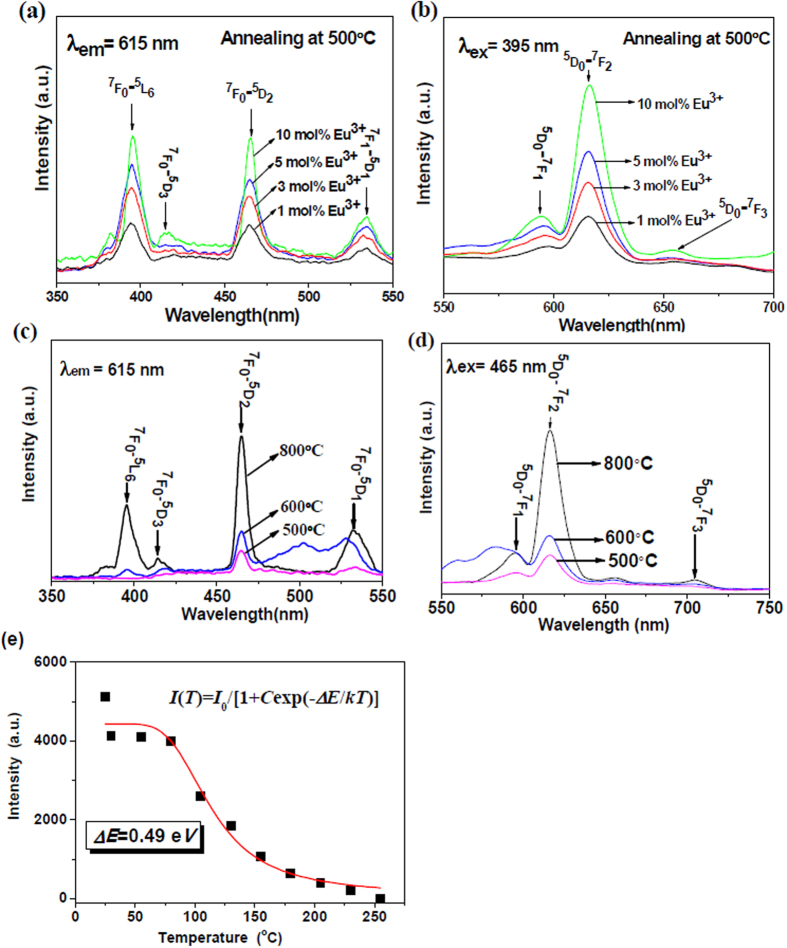
(**a**) and (**b**) are excitation (*λ*_em_ = 615 nm) and emission (*λ*_ex_ = 395 nm) spectra of well-aligned TiO_2_:Eu fibre arrays with different Eu^3+^ dopants calcined at 500 °C, respectively. (**c**) and (**d**) are excitation (*λ*_em_ = 615 nm) and emission (*λ*_ex_ = 465 nm) spectra of well-aligned TiO_2_:1.0 mol% Eu fibre arrays with different calcining temperatures, respectively. (**e**) Temperature dependence of the relative emission intensity for the Eu^3+ 5^D_0_ → ^7^F_2_ transition in the well-aligned TiO_2_:1.0 mol% Eu fibre arrays calcined at 500 °C; the scatter shows experimental data, and the solid lines are fitted functions thereto.

Figure 3(a) to (d) show the excitation and emission spectra of well-aligned TiO_2_:Eu^3+^ fibres. In the excitation spectra, the band at 200 to 280 nm was assigned to the charge transfer (CT) transition of Eu^3+^ → O^2−^. The CT band corresponds to the electronic transition from the 2*p* orbital of O^2−^ to the 4*f* orbital of Eu^3+^, and is closely related to the covalency between O^2−^ and Eu^3+^ and the coordination environment around the Eu^3+^. In the emission spectra, three peaks at 592 nm, 616 nm, and 656 nm were observed, which correspond to ^5^D_0_ → ^7^F_1_^5^, D_0_ → ^7^F_2_, and ^5^D_0_ → ^7^F_3_ transitions. As seen from the characteristic peak at 616 nm, the emission intensity increased with increasing Eu^3+^ concentration, thus implying that Eu^3+^ was not quenched while its concentration was less than 10 mol %, and agreeing with published experimental results^30^,^31^. The PL intensity of the sample increased gradually with increased calcination temperature (from 500 to 800 °C). The temperature-dependence of the emission intensity of Eu^3+^ at 616 nm, is shown in Fig. 3(e). It can be seen that the luminescence intensity decreased with increasing sample temperature, thus indicating temperature quenching occurred. This temperature quenching behaviour can be observed in most luminescent materials, but the quenching mechanisms are usually different. The increased temperature may result in an increase in the non-radiative relaxation rate for both cascade multiphonon transition and the energy transfer originating from the luminescent level^32^. In addition, the crossover process, which quenches the luminescence intensity, may occur when the temperature reaches a certain value. Usually, the fluorescence temperature quenching of Eu^3+^ doped materials follows crossover model in which the temperature dependence of fluorescence intensity can be expressed as follows^33^,^34^,


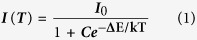


where ***I**(**T***) presents fluorescence intensity at temperature ***T***; ***I***_0_ is the initial intensity at temperature closed to 0 K; ***ΔE*** stands for the activation energy; ***k*** is Boltzmann’s constant; ***C*** is a constant for a certain system. To examine the temperature quenching mechanism of Eu^3+^ fluorescence, Eq. (1) was fit to the data in Fig. 3(e), and it can be seen Eq. (1) can fit well with the data. In the fitting process, the ***ΔE***was confirmed to be 0.49 eV which in reasonable agreement with the results reported in Eu^3+^ doped other materials^35^,^36^. These results reflected that the fluorescence temperature quenching was evoked by crossover process.

**Figure 4:**
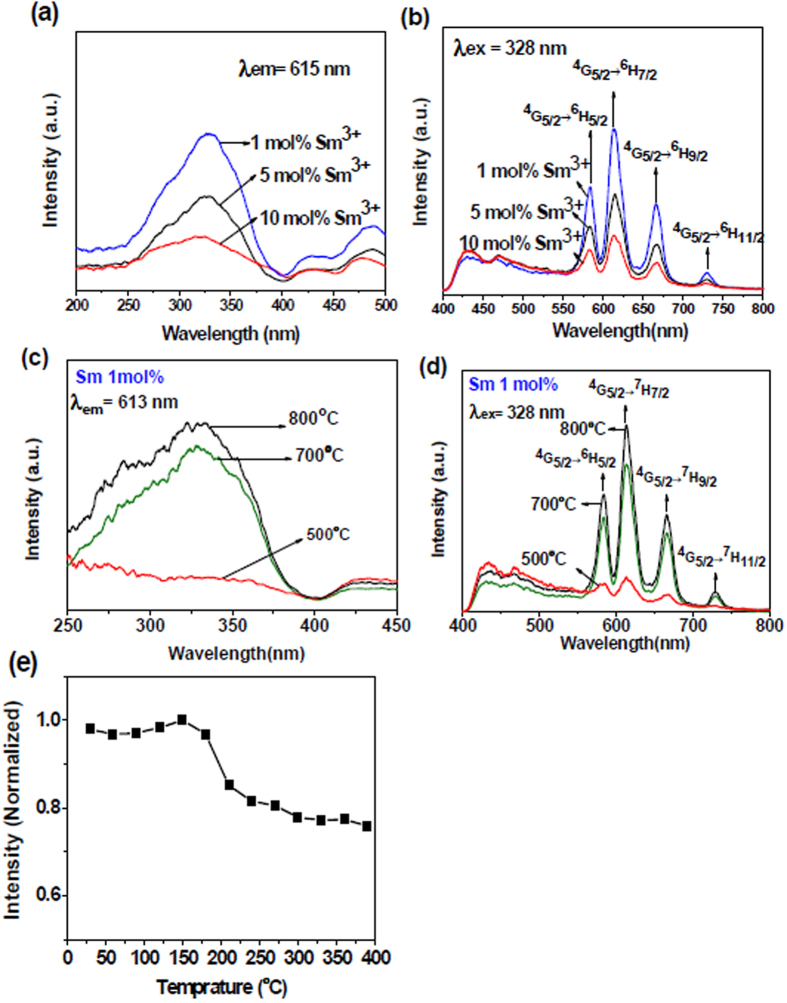
(**a**) and (**b**) are excitation (*λ*_em_ = 615 nm) and emission (*λ*_ex_ = 328 nm) spectra of well-aligned TiO_2_:Sm fibre arrays with variable Sm^3+^ dopant concentrations calcined at 600 °C, respectively. (**c**) and (**d**) are excitation (*λ*_em_ = 613 nm) and emission (*λ*_ex_ = 328 nm) spectra of well-aligned TiO_2_:1.0 mol% Sm fibre arrays with different calcining temperatures, respectively. (**e**) Temperature dependence of the relative emission intensity for Sm^3+ 4^G_5/2_ → ^6^H_7/2_ transition in the well-aligned TiO_2_:1.0 mol% Sm fibre arrays calcined at 600 °C.

Figure 4 (a) to (d) show the excitation (*λ*_em_ = 615 nm) and emission (*λ*_ex_ = 328 nm) spectra of well-aligned TiO_2_:Sm nanofibres. For the excitation spectra, one intense broad excitation band centred at 328 nm was observed, which corresponded to the anatase TiO_2_ host absorption. This evinced an effective energy transfer from the TiO_2_ host to the Sm^3+^ ions. Three obvious emission peaks centred at 580, 615, and 660 nm were observed in the emission spectra, which can be ascribed to the transitions from the excited state ^4^G_5/2_ to the ^6^H_5/2_, ^6^H_7/2_, and ^6^H_9/2_ states, respectively. As seen from the characteristic peak at 615 nm, the emission intensity decreased with increasing Sm^3+^ concentration; however, the emissions of the TiO_2_:1 mol % Sm^3+^ samples, upon increasing the calcining temperature from 500 to 800 °C showed an increasing temperature-dependence (Fig. 4(e)) on the integrated luminescence intensity for the ^4^G_5/2_ → ^6^H_7/2_ transition on the TiO_2_:1 mol % Sm^3+^ sample. It can be seen that the integrated emission intensity keeps almost unchanged within the temperature range of 30–180 °C, and then decreases with increasing sample temperature. That is to say, the thermal quenching occurred at temperatures greater than 180 °C. The emission intensity of Sm^3+^ decreased to approximately 85% of its low-temperature level at 400 °C. It should also be mentioned that the quenching process, namely the variation trend of integrated luminescence intensity toward temperature, is extremely different from the one for TiO_2_:Eu^3+^. This is because of the fact that the excited state ^4^G_5/2_ is much nearer to the neighboring lower state, thus the nonradiative transition can not be omitted at higher temperature. This fact means that the fluorescence temperature quenching can not be simply explained by crossover process.

**Figure 5:**
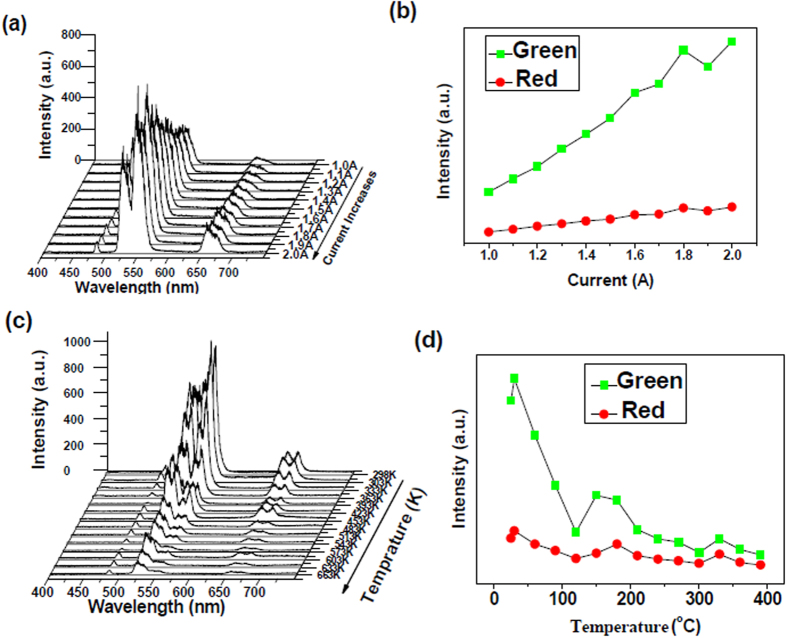
(**a**) Up-conversion emission spectra of TiO_2_:1.0 mol% Er fibre arrays under excitation at 980 nm; (**b**) Corresponding dependences of green and red up-conversion emission intensities on the laser working current. (**c**) Emission spectra of TiO_2_:1.0 mol% Er fibre arrays measured at various temperatures; (**d**) Corresponding dependences of green and red up-conversion intensities on sample temperature.

Figure 5 (a) to (d) show the emission (*λ*_ex_ = 328 nm) spectra of well-aligned TiO_2_:Er nanofibres under excitation at 980 nm. The strong, weak, and very weak emission bands located at 550 nm, 525 nm (green region), and 655 nm (red region) were observed, and their intensities monotonically increased with increasing laser working current. The strong 550 nm emission bands, and the weak 525 nm bands in the green region, corresponded to the ^4^S_3/2_ → ^4^I_15/2_ and ^2^H_11/2_ → ^4^I_15/2_ transitions, respectively, while the very weak emission 655 nm bands in the red region corresponded to the ^4^F_9/2_ → ^4^I_15/2_ transition. The green UC emissions were dominant, and the green emission was much more intense than that in the red bandwidth region once the laser working current had been increased; however, the green emission was more rapidly decreased than that in the red region with increasing temperature (<120 °C). In comparison with Figs 3(e), 4(e) and 5(d) showed more complicated temperature-dependent fluorescence intensity. Obviously, this temperature of Er^3+^ doped TiO_2_ fiber arrays impossible obeys the crossover model since in the UC luminescence process many metastable levels of Er^3+^ were involved into the population process, and the populations of all these levels related to the temperature, thus the simple potential barrier model for crossover process can not explain it.

## Conclusions

The continuous, highly oriented, TiO_2_:Ln^3+^ (Ln = Eu, Sm, or Er) fibre arrays with linear alignment were successfully prepared by use of an improved electrospinning method. The advantages of the modified method were as follows: (1) exploitation of the viscoelasticity of ultrahigh molecular weight polymer solutions to eliminate the occurrence of bending instability, will lead to a synthetic process that is simple, rapid, efficient, and cost-effective; (2) it can be used to obtain electrospun fibre arrays measuring at least 300 mm in length; (3) the need for a high-speed rotating drum, a manipulating or electrical field, and an expensive collector were all obviated; (4) it provided a continuous fibre-aligning capability and can offer the ability for mass-scale manufacture on a commercial scale. Their luminescence properties were also studied. These results shown that the emission intensity of TiO_2_:Eu^3+^ fibre arrays were enhanced with increasing Eu^3+^ concentration. On the contrary, the emission intensity of TiO_2_:Sm^3+^ fibre arrays decreased with increasing Sm^3+^ concentration. The emission intensities of TiO_2_:Er^3+^ fibre arrays increased monotonically with increasing laser working current. In addition, these TiO_2_:Ln^3+^ fibre arrays presented typical temperature quenching behaviour. Overall, this technique, and its extension to other metal oxides or metal ion-doped oxides, will open up novel routes to the functionalisation of surfaces that can enable the fabrication of new types of optical, electric, magnetic, and tissue engineering devices.

## Additional Information

**How to cite this article**: Yu, H. *et al*. Ultralong well-aligned TiO_2_:Ln^3+^ (Ln = Eu, Sm, or Er) fibres prepared by modified electrospinning and their temperature-dependent luminescence. *Sci. Rep.* **7**, 44099; doi: 10.1038/srep44099 (2017).

**Publisher's note:** Springer Nature remains neutral with regard to jurisdictional claims in published maps and institutional affiliations.

## Supplementary Material

Supplementary Information

## References

[b1] Jen H. P., Lin M. H., Li L. L., Wu H. P. & Huang W. K. High-performance large-scale flexible dye-sensitized solar cells based on anodic TiO_2_ nanotube arrays. ACS Appl. Mater. & Inter. 5, 10098–10104 (2013). 10.1021/am402687j 24050628

[b2] Liu S., Hu J. J., Yan N. F., Pan G. L. & Li G. R. Aluminum storage behavior of anatase TiO_2_ nanotube arrays in aqueous solution for aluminum ion batteries. Energy & Environ. Sci. 5, 9743–9746 (2012).

[b3] Liu Z., Zhang X., Nishimoto S., Jin M. & Tryk D. A. Highly ordered TiO_2_ nanotube arrays with controllable length for photoelectrocatalytic degradation of phenol. J. Phys. Chem. C 112, 253–259 (2008).

[b4] Shao Z., Zhu W., Li Z., Yang Q. & Wang G. One-step fabrication of CdS nanoparticle-sensitized TiO_2_ nanotube arrays via electrodeposition. J. Phys. Chem. C 116, 2438–2442 (2012).

[b5] Song J., Xu L., Xing R., Qin W. & Dai Q. Ag nanoparticles coated NiO nanowires hierarchical nanocomposites electrode for nonenzymatic glucose biosensing. Sens. Actuators, B 182, 675–681 (2013).

[b6] Xu L., Dong B., Wang Y., Bai X. & Liu Q. Electrospinning preparation and room temperature gas sensing properties of porous In_2_O_3_ nanotubes and nanowires. Sens. Actuators, B 147, 531–538 (2010).

[b7] Qin W., Xu L., Song J., Xing R. & Song H. Highly enhanced gas sensing properties of porous SnO_2_-CeO_2_ composite nanofibers prepared by electrospinning. Sens. Actuators, B 185, 231–237 (2013).

[b8] Zhou C., Xu L., Song J., Xing R. & Xu S. Ultrasensitive non-enzymatic glucose sensor based on three-dimensional network of ZnO-CuO hierarchical nanocomposites by electrospinning. Sci. Rep-UK 4, 7382 (2014). 10.1038/srep07382 PMC4260231 25488502

[b9] Xing R., Xu L., Song J., Zhou C. & Li Q. Preparation and gas sensing properties of In_2_O_3_/Au nanorods for detection of volatile organic compounds in exhaled breath. Sci. Rep-UK 5, 10717 (2015). 10.1038/srep10717 PMC5377237 26030482

[b10] Paulose M., Shankar K., Yoriya S., Prakasam H. E. & Varghese O. K. Anodic growth of highly ordered TiO_2_ nanotube arrays to 134 μm in length. J. Phys. Chem. B 110(33), 16179–16184 (2006). 16913737 10.1021/jp064020k

[b11] Zhou H. & Zhang Y. Electrochemically self-doped TiO_2_ nanotube arrays for supercapacitors. J. Phys. Chem. C 118, 5626–5636 (2014).

[b12] Yu Z., Qu X., Yang W., Peng J. & Xu Z. Hydrothermal synthesis and memristive switching behaviors of single-crystalline anatase TiO_2_ nanowire arrays. J. Alloy. Compd. 15, 294–300 (2016).

[b13] Dersch R., Liu T., Schaper A. K., Greiner A. & Wendorff J. H. Electrospun nanofibers: Internal structure and intrinsic orientation. J. Polym. Sci. Part B. 41, 545–553 (2003).

[b14] Liu Z., Li X., Yang Y., Zhang K. & Wang X. Control of structure and morphology of highly aligned PLLA ultrafine fibers via linear-jet electrospinning, Polymer 54, 6045–6051 (2013).

[b15] Zhou Q., Bao M., Yuan H., Zhao S. & Dong W. Implication of stable jet length in electrospinning for collecting well-aligned ultrafine PLLA fibers, Polymer 54, 6867–6876 (2013).

[b16] Theron A., Zussman E. & Yarin A. L. Formation of nanofiber crossbars in electrospinning. Appl. Phys. Lett. 82, 973 (2003).

[b17] Theron A., Zussman E. & Yarin A. L. Electrostatic field-assisted alignment of electrospun nanofibres. Nanotechnology 12, 3–7 (2001).

[b18] Li D. & Xia Y. Electrostatic field-assisted alignment of electrospun nanofibres. Nano. Lett. 3, 555–560 (2003).

[b19] Xu S., Dong B., Zhou D., Yin Z. & Cui S. Paper-based upconversion fluorescence resonance energy transfer biosensor for sensitive detection of multiple cancer biomarkers. Sci. Rep-UK 6, 23406 (2016). 10.1038/srep23406 PMC4802215 27001460

[b20] Dong B., Liu D. P., Wang X. J., Yang T. & Miao S. M. Optical thermometry through infrared excited green upconversion emissions in Er^3+^-Yb^3+^ codoped Al_2_O_3_. Appl. Phys. Lett. 90, 181117 (2007).

[b21] Dong B., Hua R. N., Cao B. S., Li Z. P. & He Y. Y. Size dependence of the upconverted luminescence of NaYF_4_:Er,Yb microspheres for use in ratiometric thermometry. Phys. Chem. Chem. Phys. 16, 20009–20012 (2014). 25123272 10.1039/c4cp01966k

[b22] Dong B., Cao B., He Y., Liu Z. & Li Z. Temperature sensing and *in vivo* imaging by molybdenum sensitized visible upconversion luminescence of rare-earth oxides. Adv. Mater. 24, 1987–1993 (2012). 22422477 10.1002/adma.201200431

[b23] Liu K., Zhang Z., Shan C., Feng Z. & Li J. A flexible and superhydrophobic upconversionluminescence membrane as an ultrasensitive fluorescence sensor for single droplet detection. Light-Sci. Appl. 5, e16136 (2016). 10.1038/lsa.2016.136 PMC6059937 30167183

[b24] Yu H., Li T., Chen B., Wu Y. & Li Y. Preparation of aligned Eu(DBM)_3_phen/PS fibers by electrospinning and their luminescence properties. J. Colloid. Interf. Sci. 400, 175–180 (2013). 10.1016/j.jcis.2013.03.017 23578517

[b25] Huang Z., Zhang Y. Z., Kotaki M. & Ramakrishn S. A review on polymer nanofibers by electrospinning and their applications in nanocomposites. Compos. Sci. Technol. 63, 2223–2253 (2003).

[b26] Luo G., Teh K. S., Liu Y., Zang X. & Wen Z. Direct-write, self-aligned electrospinning on paper for controllable fabrication of three-dimensional structures. ACS Appl. Mater. Inter. 7, 27765–27770 (2015). 10.1021/acsami.5b08909 26592741

[b27] Valente T. A., Silva D. M., Gomes P. S., Fernandes M. H. & Santos J. D. Effect of sterilization methods on electrospun poly(lactic acid) (PLA) fiber alignment for biomedical applications. ACS Appl. Mater. Inter. 8, 3241–3249 (2016). 10.1021/acsami.5b10869 26756809

[b28] Chew S. Y., Mi R., Hoke A. & Leong K. W. The effect of the alignment of electrospun fibrous scaffolds on Schwann cell maturation. Biomaterials 29, 653–661 (2008). 17983651 10.1016/j.biomaterials.2007.10.025 PMC2713097

[b29] Dong G., Xiao X., Chi Y., Qian B. & Liu X. Polarized luminescence properties of TiO_2_:Sm^3+^ microfibers and microbelts prepared by electrospinning. J. Phys. Chem. C 113, 9595–9600 (2009).

[b30] Yi S., Bae J. S., Moon B. K., Jeong J. H. & Kim J. H. Highly enhanced luminescence of nanocrystalline TiO_2_:Eu^3+^ phosphors. Opt. Mater. 28, 610–614 (2006).

[b31] Leostean C., Stefan M., Pana O., Cadis A. I. & Suciu R. C. Properties of Eu doped TiO_2_ nanoparticles prepared by using organic additives. J. Alloy. Compd. 575, 29–39 (2013).

[b32] Tian Y., Chen B. J., Hua R. N., Sun J. S. & Cheng L. H. Optical transition, electron-phonon coupling and fluorescent quenching of La_2_(MoO4)_3_:Eu^3+^ phosphor. J. Appl. Phys. 109, 053511 (2011).

[b33] Van Uitert L. G. Characterization of energy transfer interactions between rare earth ions. J. Electrochem. Soc. 114, 1048–1053 (1967).

[b34] Struck C. W. & Fonger W. H. Quantum-mechanical treatment of Eu^3+^ 4f → 4f and 4f? charge-transfer-state transitions in Y2O_2_S and La2O_2_S. J. Chem. Phys. 64, 1784–1790 (1976).

[b35] Tian B., Chen B., Tian Y., Li X. & Zhang J. Excitation pawthway and temperature dependent luminescence in color tunable Ba_2_Gd_8_Zn_4_O_21_:Eu^3+^ phosphors. J. Mater. Chem. C 1, 2338–2344 (2013).

[b36] Tian Y., Chen B., Hua R., Yu N. & Liu B. Self-assembled 3D flower-shaped NaY(WO_4_)_2_:Eu^3+^ microarchitectures: Microwave-assisted hydrothermal synthesis, growth mechanism and luminescence properties. CrystEngComm. 14, 1760–1769 (2012).

