# Supplementary material for: Ultralong well-aligned TiO2:Ln3+ (Ln = Eu, Sm, or Er) fibres prepared by modified electrospinning and their temperature-dependent luminescence
Source: Sci Rep. 2017 Mar 7;7:44099. doi: 10.1038/srep44099 (PMC5339778; doi:10.1038/srep44099)
Supplement: Supplementary Information [file srep44099-s1.doc]

**Supplementary Information**

**Ultralong well-aligned TiO_2_:Ln^3+^ (Ln = Eu, Sm, or Er) fibres prepared by modified electrospinning and their temperature-dependent luminescence**

Hongquan Yu^1^,*, Yue Li^1^, Yang Song^1^, Yanbo Wu^1^, Xijie Lan^1^, Shimin Liu^1^, Yanning Tang^1^, Shasha Xu^1^ and Baojiu Chen^2^,*

1. *College of Environmental and Chemical Engineering,* *Dalian Jiaotong University, Dalian, Liaoning 116028, P.R. China*

2. *Department of Physics, Dalian Maritime University, Dalian, Liaoning 116026, P.R. China.*

*Corresponding author: **Hongquan Yu** (H. Yu)

Address: Dalian Jiaotong University, No. 794, Huanghe Road, Dalian 116028, People’s Republic of China.

E-mail: [yuhq7808@djtu.edu.cn](mailto:yuhq7808@djtu.edu.cn) (H. Yu); [bjchen@dlmu.edu.cn](mailto:bjchen@dlmu.edu.cn) (B. Chen)

Tel: +86-411-84106809 Fax: +86-411-84106890

**Supplementary Figure.1** Schematic of the electrospinning set-up.


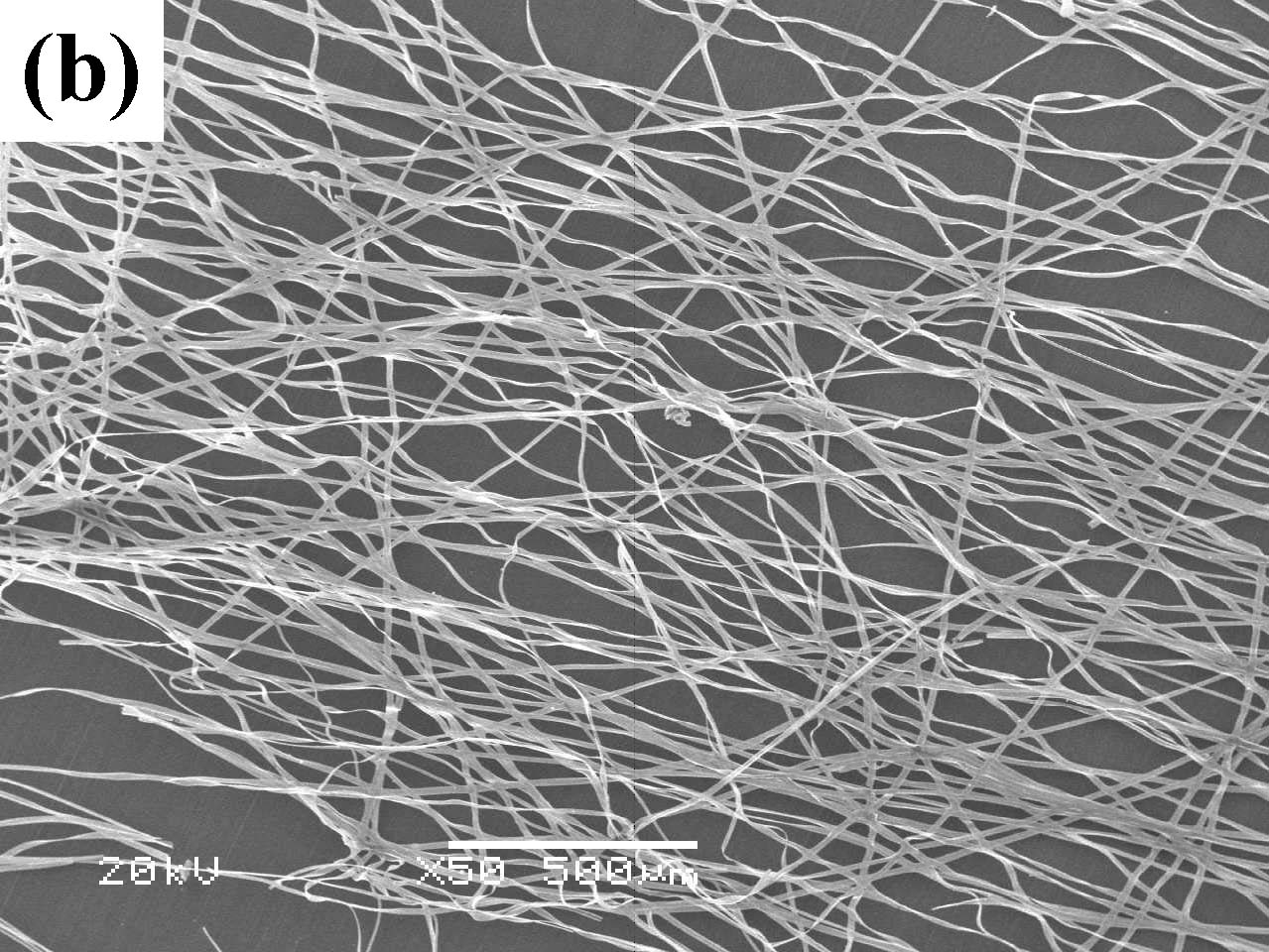

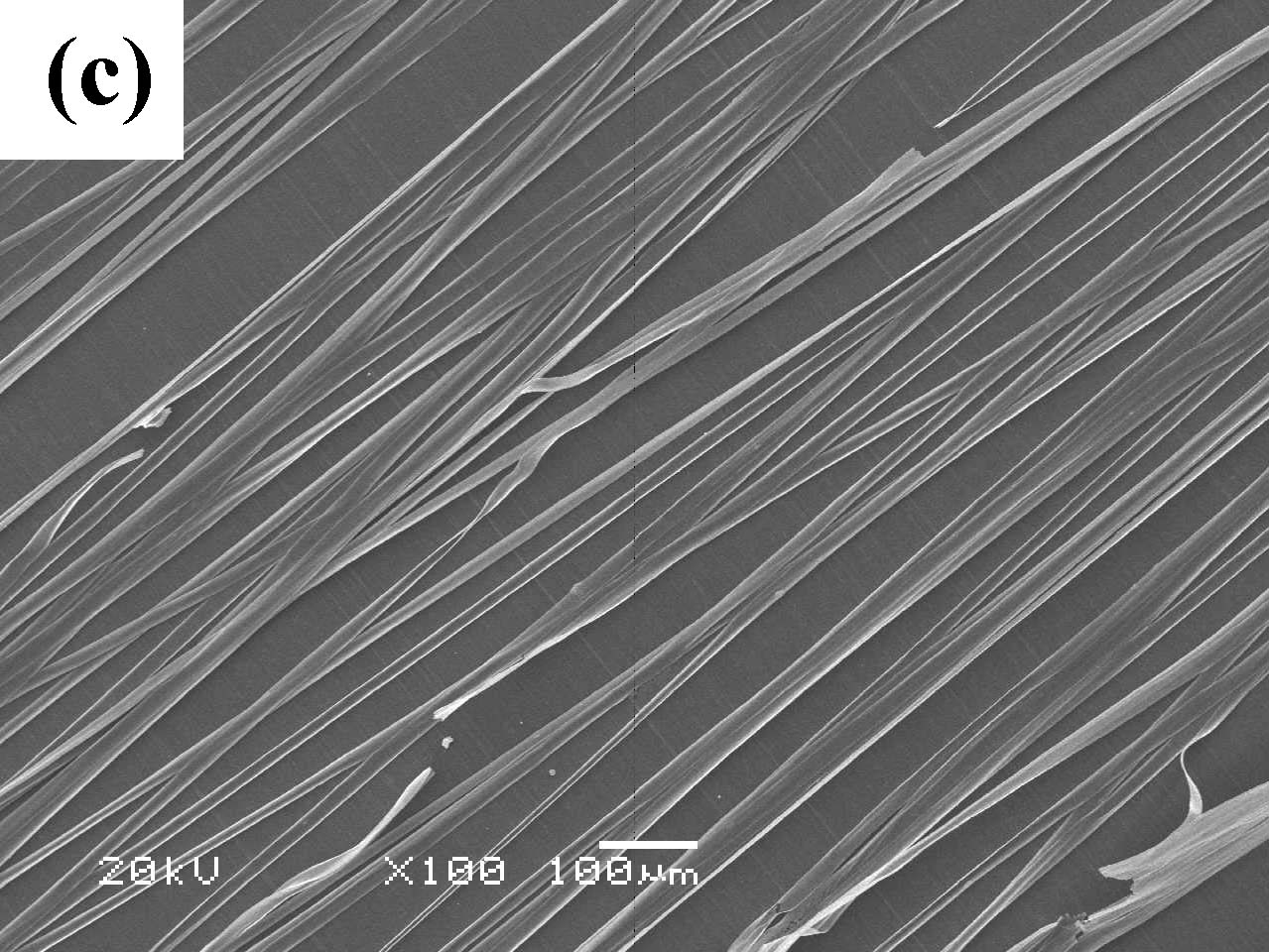

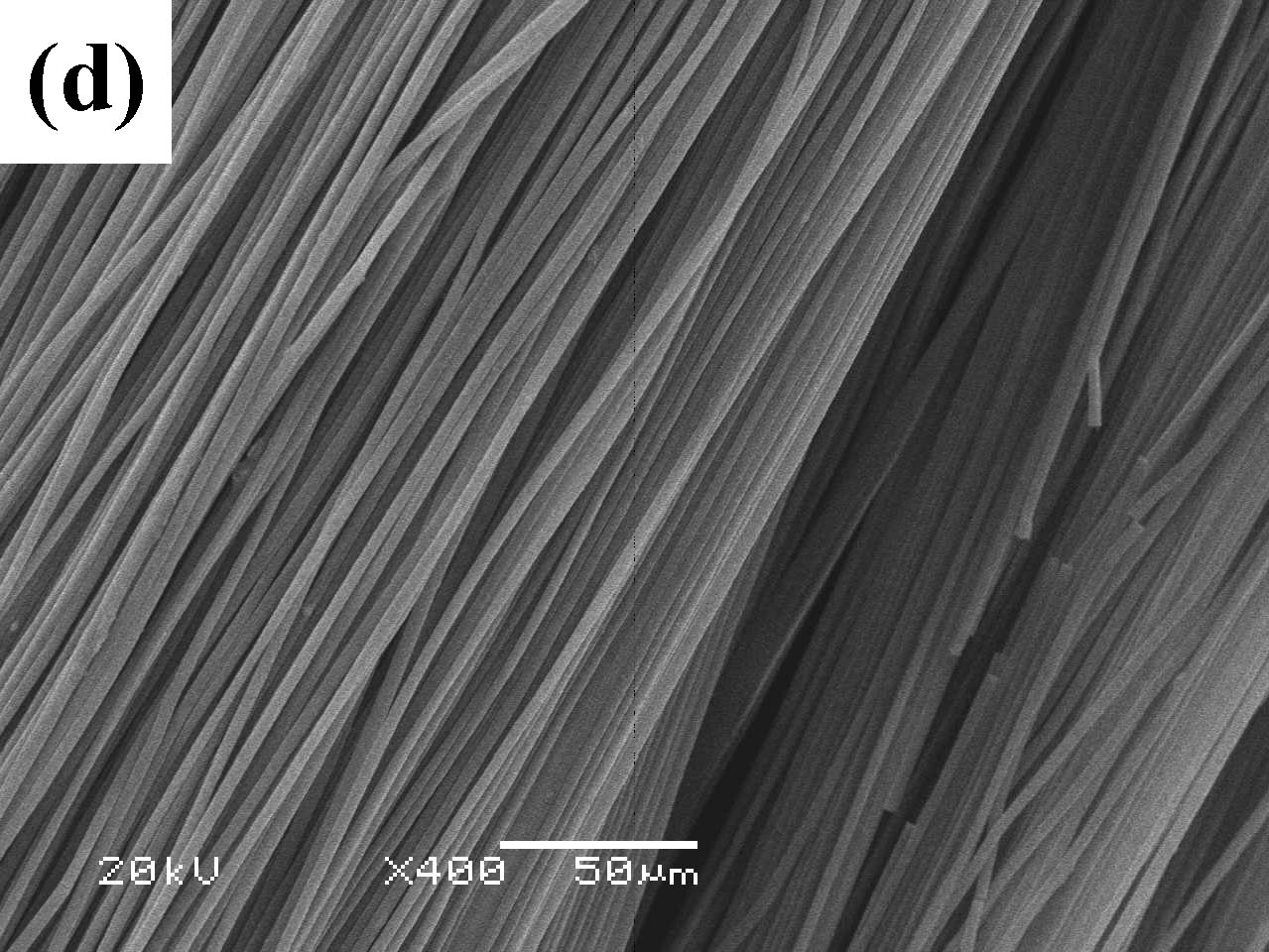

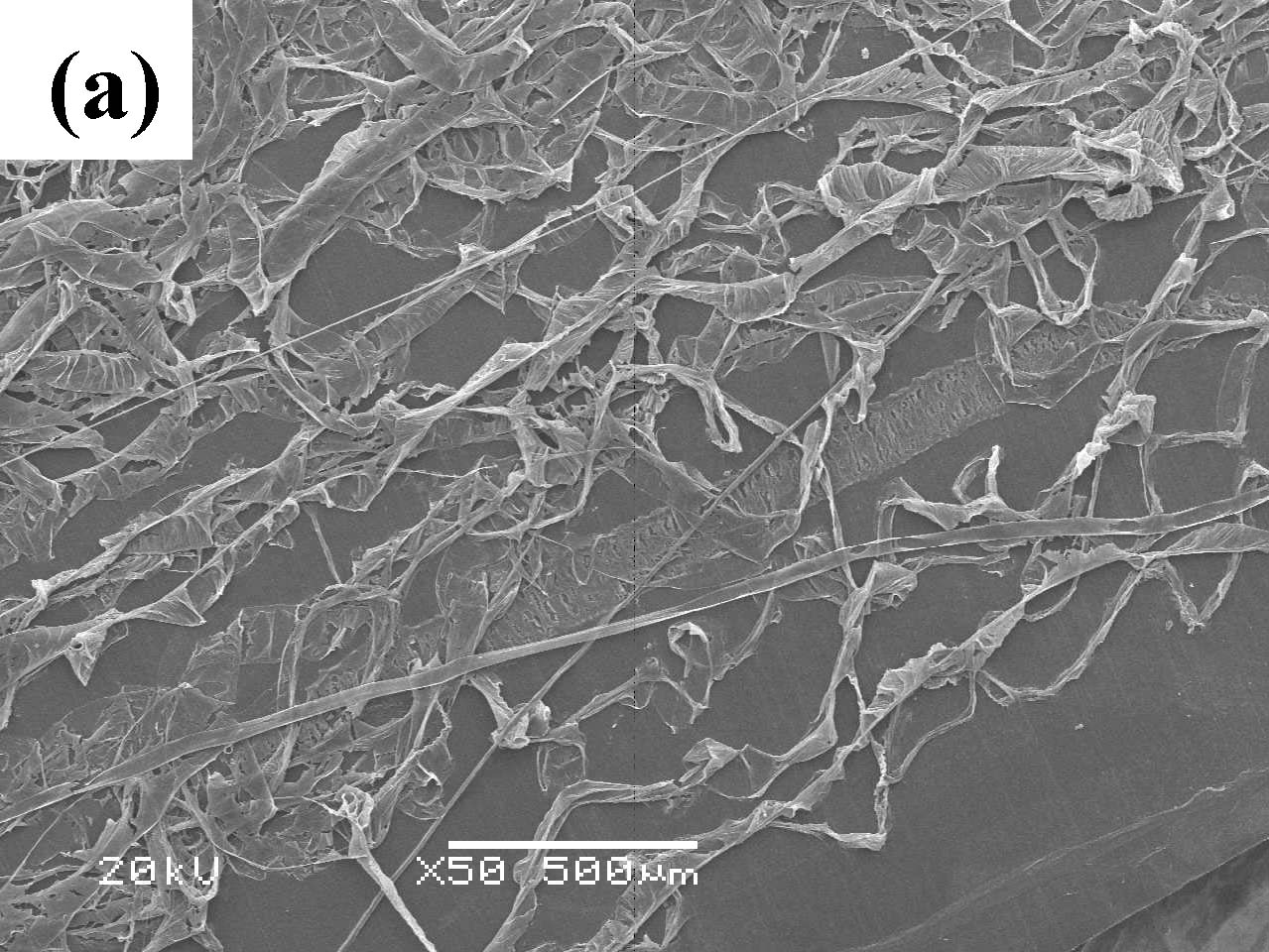

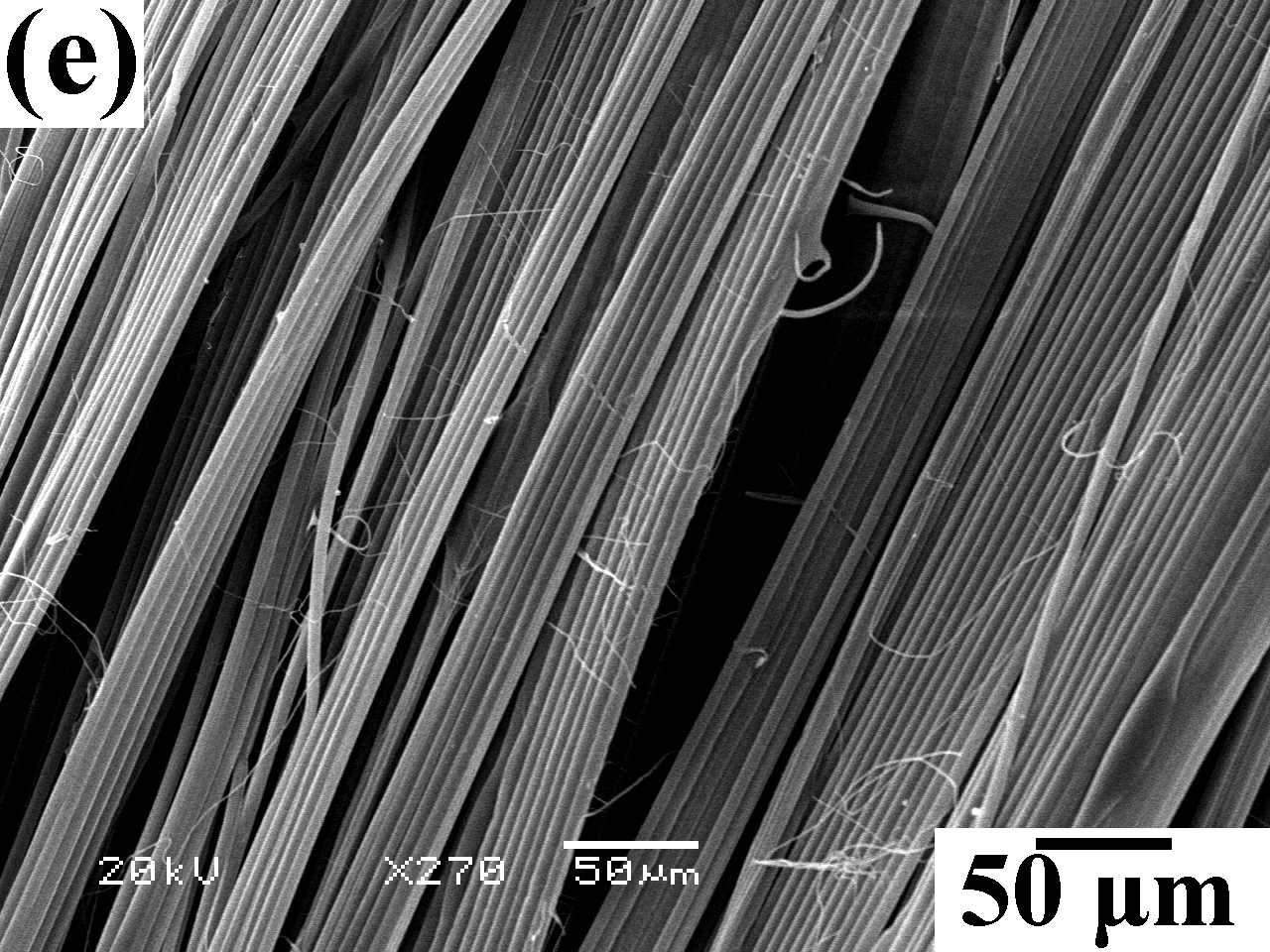

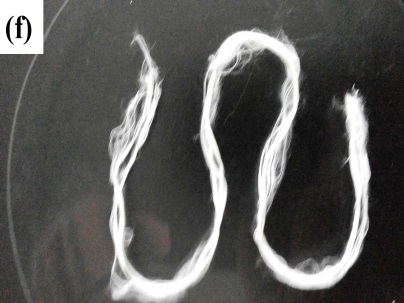


**Supplementary Figure.2** SEM images of precursor fibres of TiO_2_ on the drum with various rates of rotation: (a) 500 rpm, (b) 800 rpm, (c) 1100 rpm, and (d) 1400 rpm. (e) shows a photograph of the well-aligned precursor TiO_2_ fibres. Here, 1 rpm was equivalent to a linear velocity of 0.0067 m/s. The concentration of ultrahigh molecular weight PEO was fixed at 2.5 wt %. The applied voltage was 8.0 kV and the distance between the spinneret tip and collector was 200 mm.


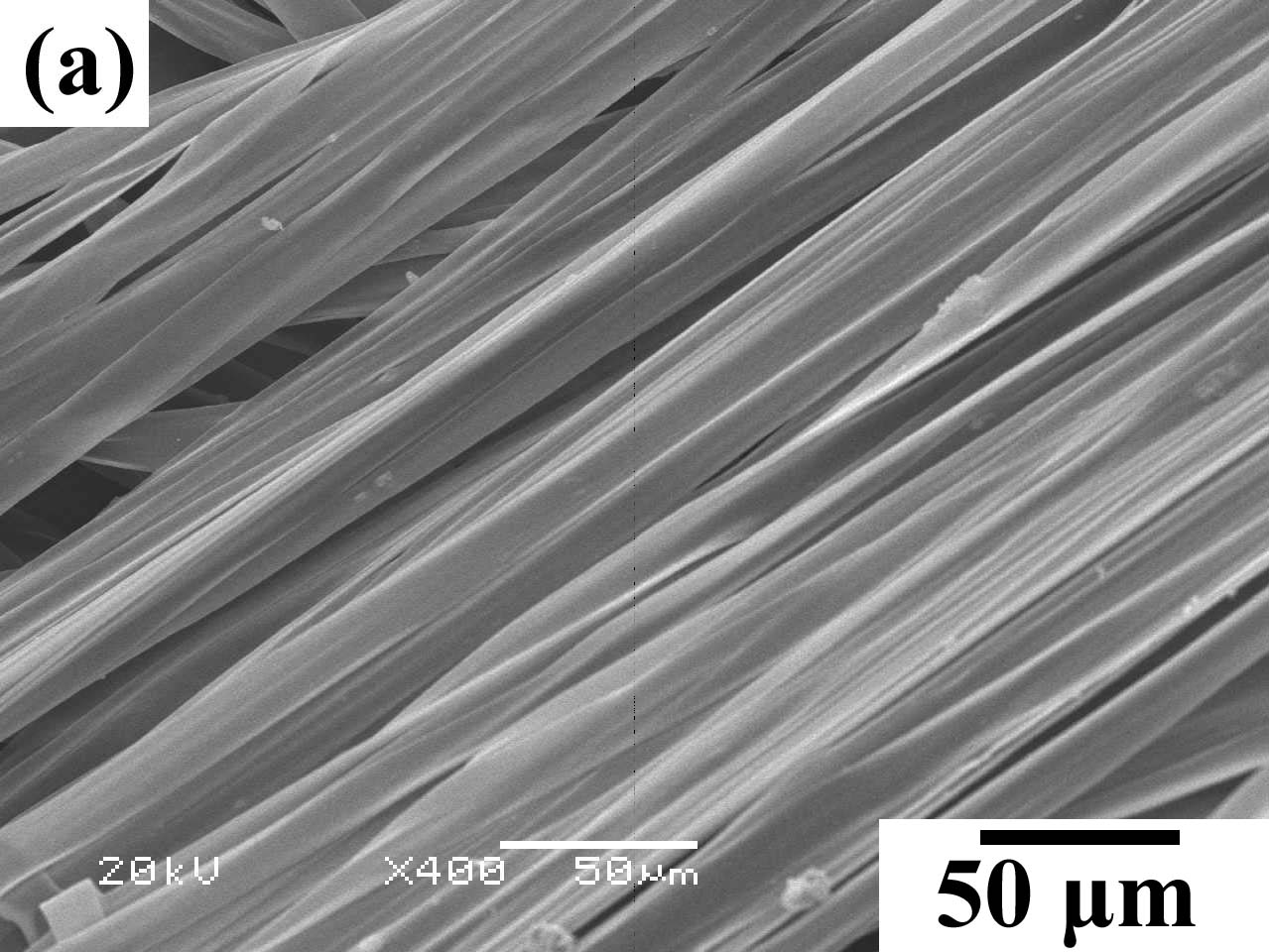

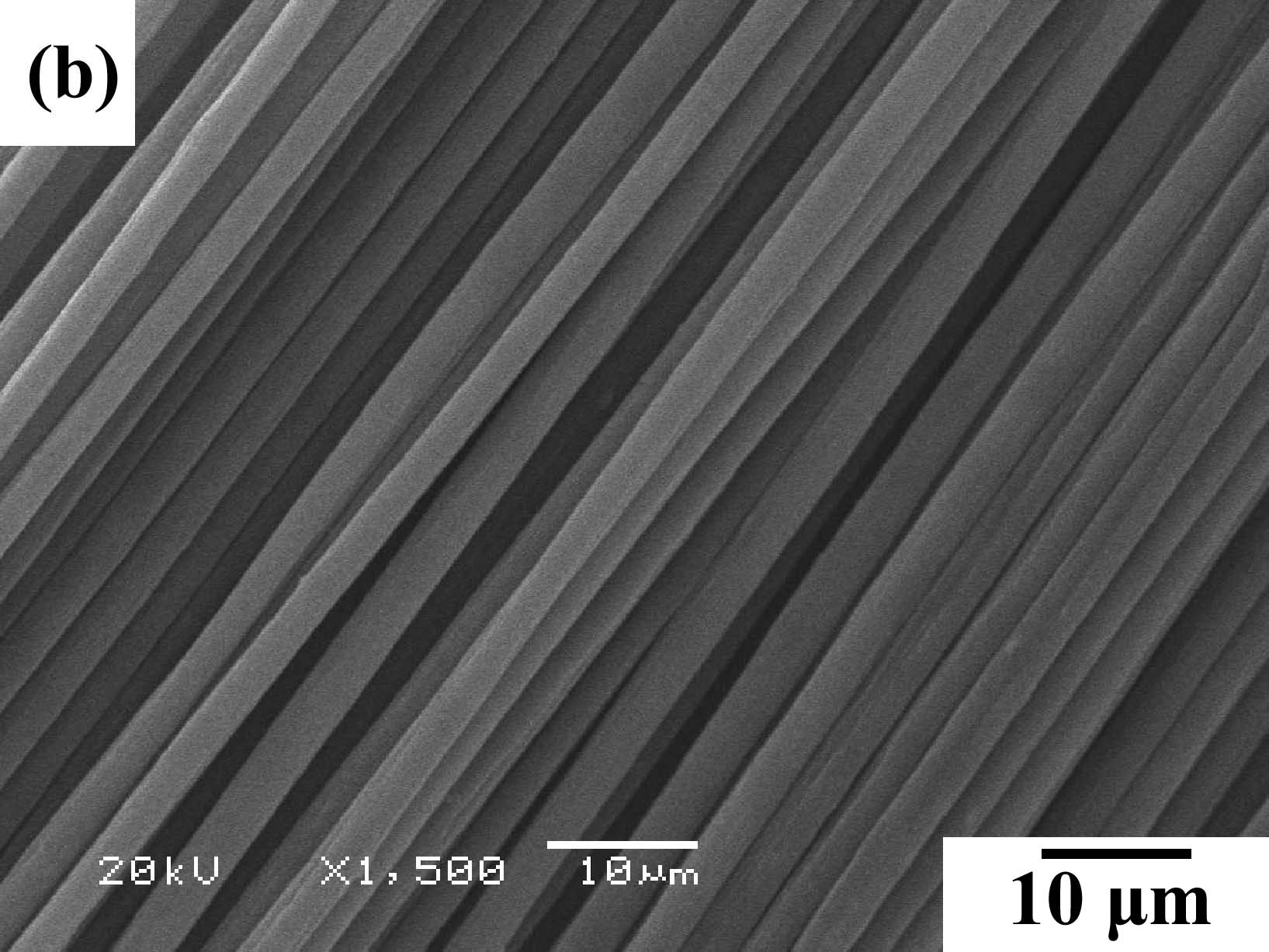

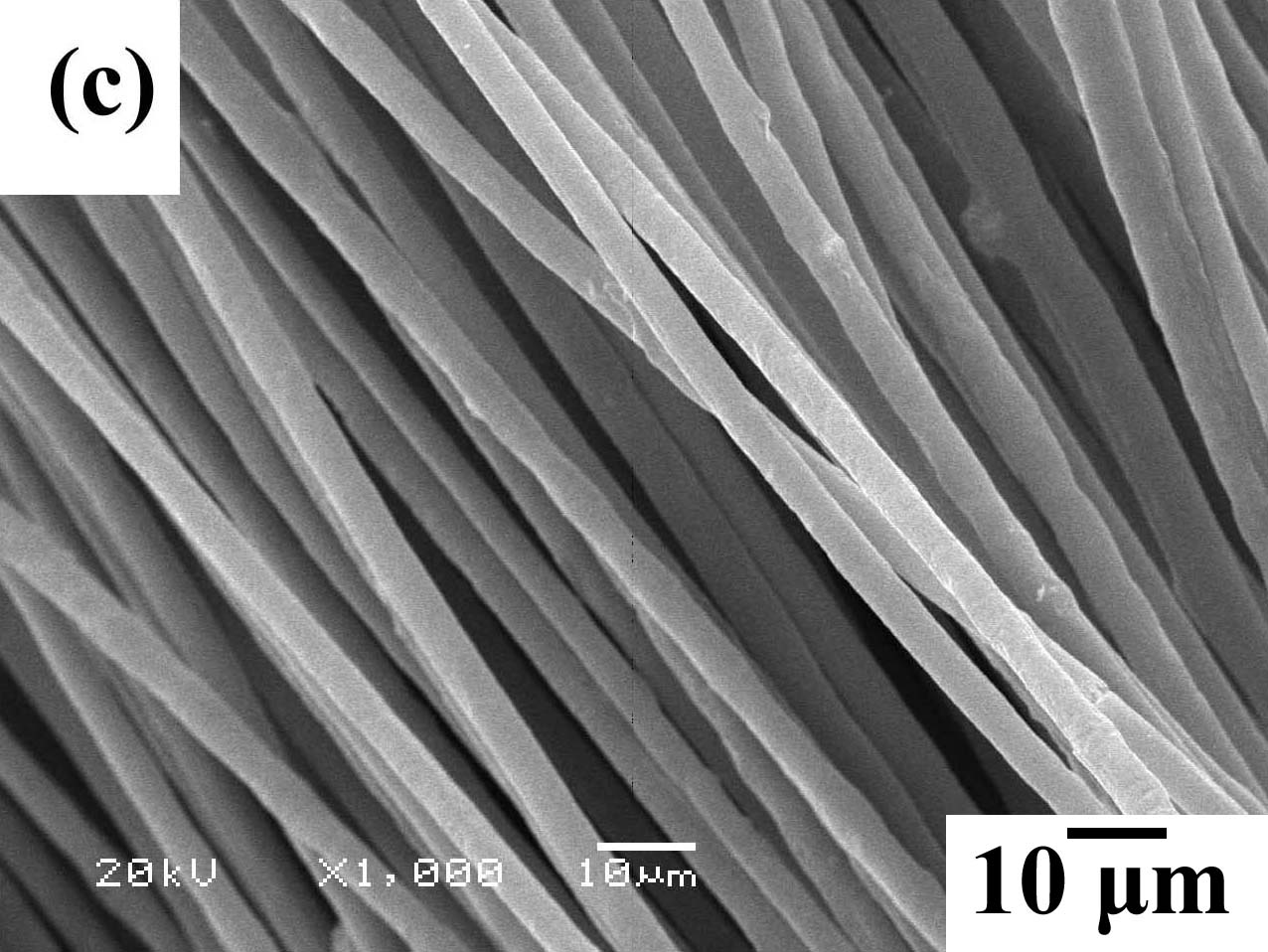

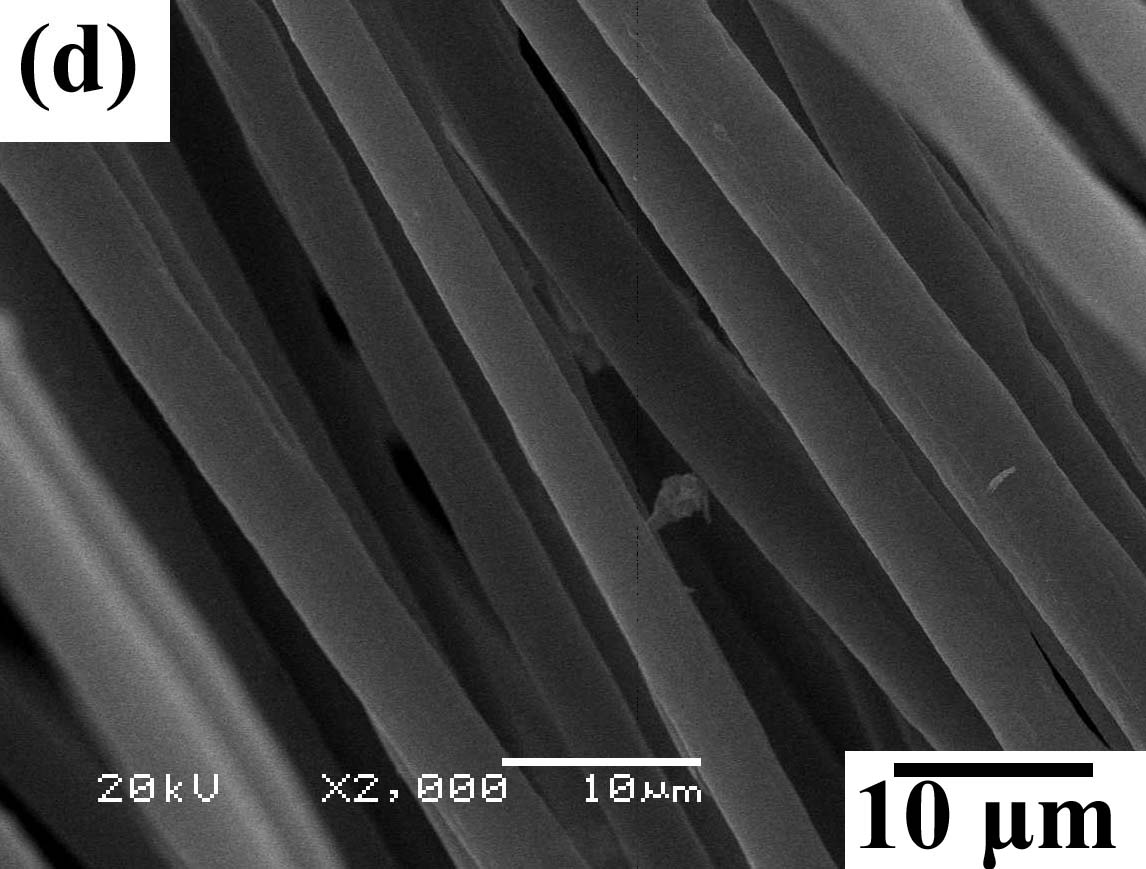


**Supplementary Figure.3** SEM images of the well-aligned precursor TiO_2_ fibres obtained from different concentration PEO: (a) 1.0 wt%; (b) 2.0 wt %; (c) 2.5 wt %; (d) 3.0 wt %. The concentration of Ti(OC_4_H_9_)_4_) was fixed at 8.0 wt %. The rate of rotation of the collecting drum was fixed at 1400 rpm. Here, 1 rpm was equivalent to a linear velocity of 0.0067 m/s.


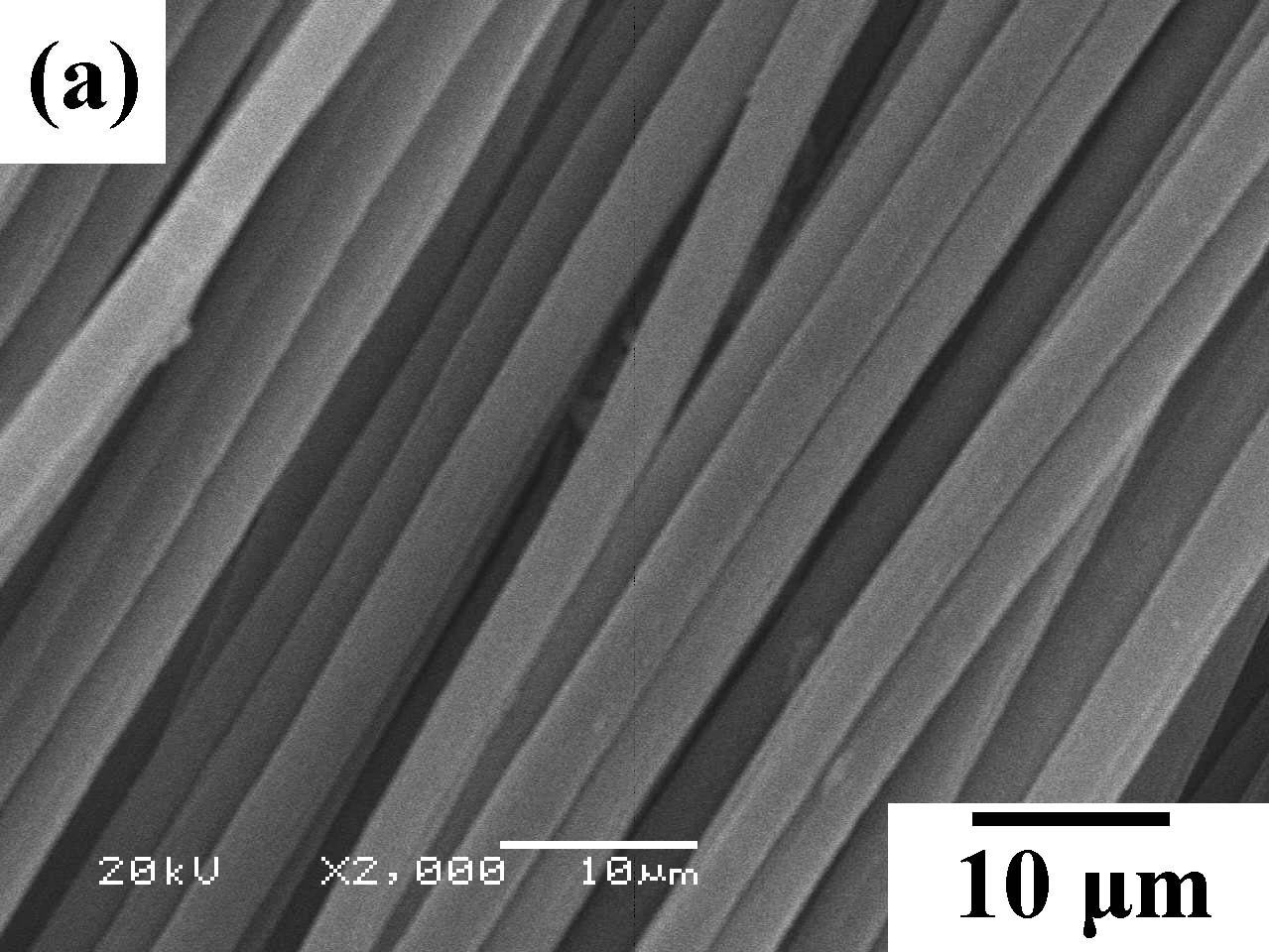

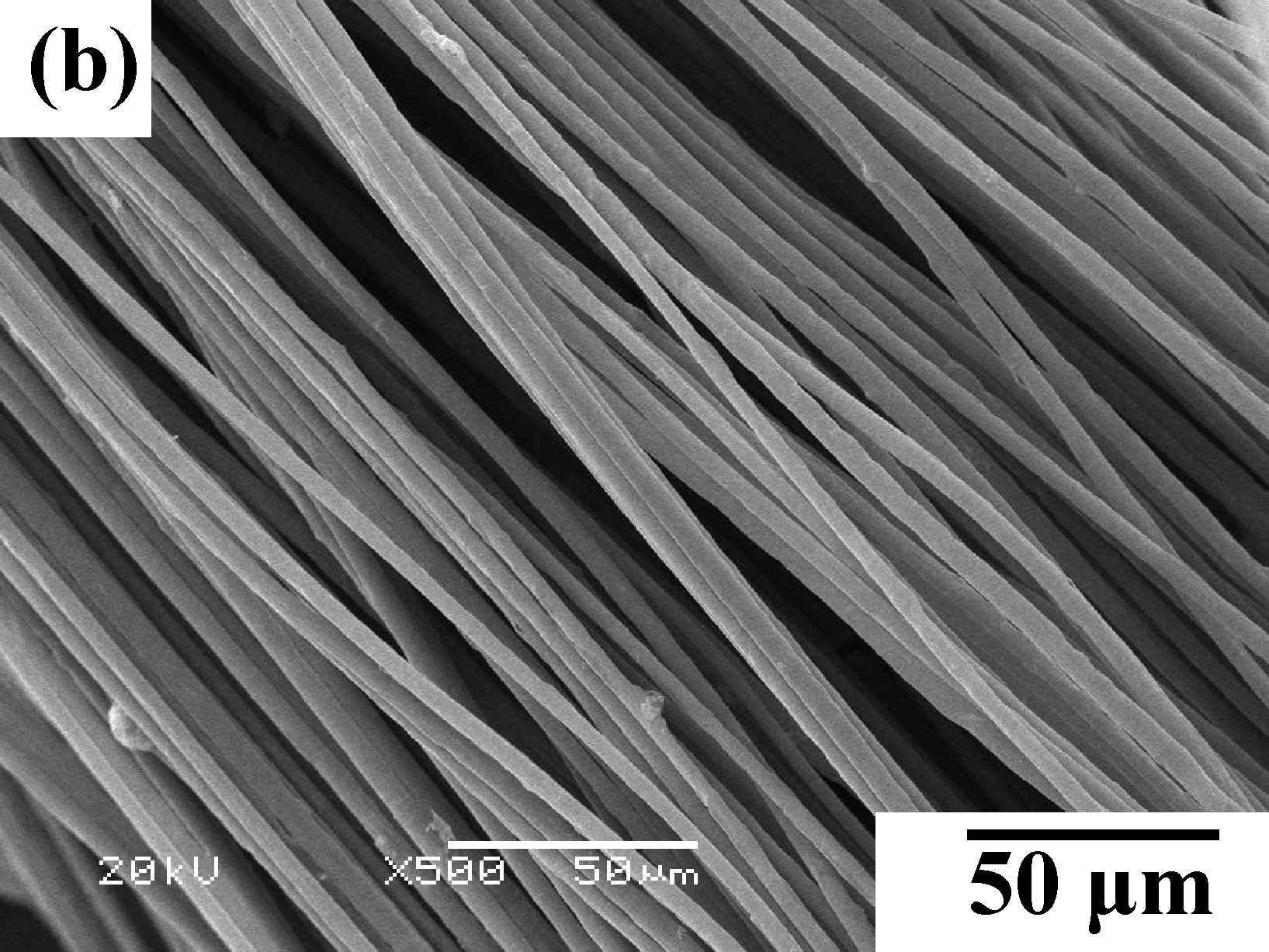

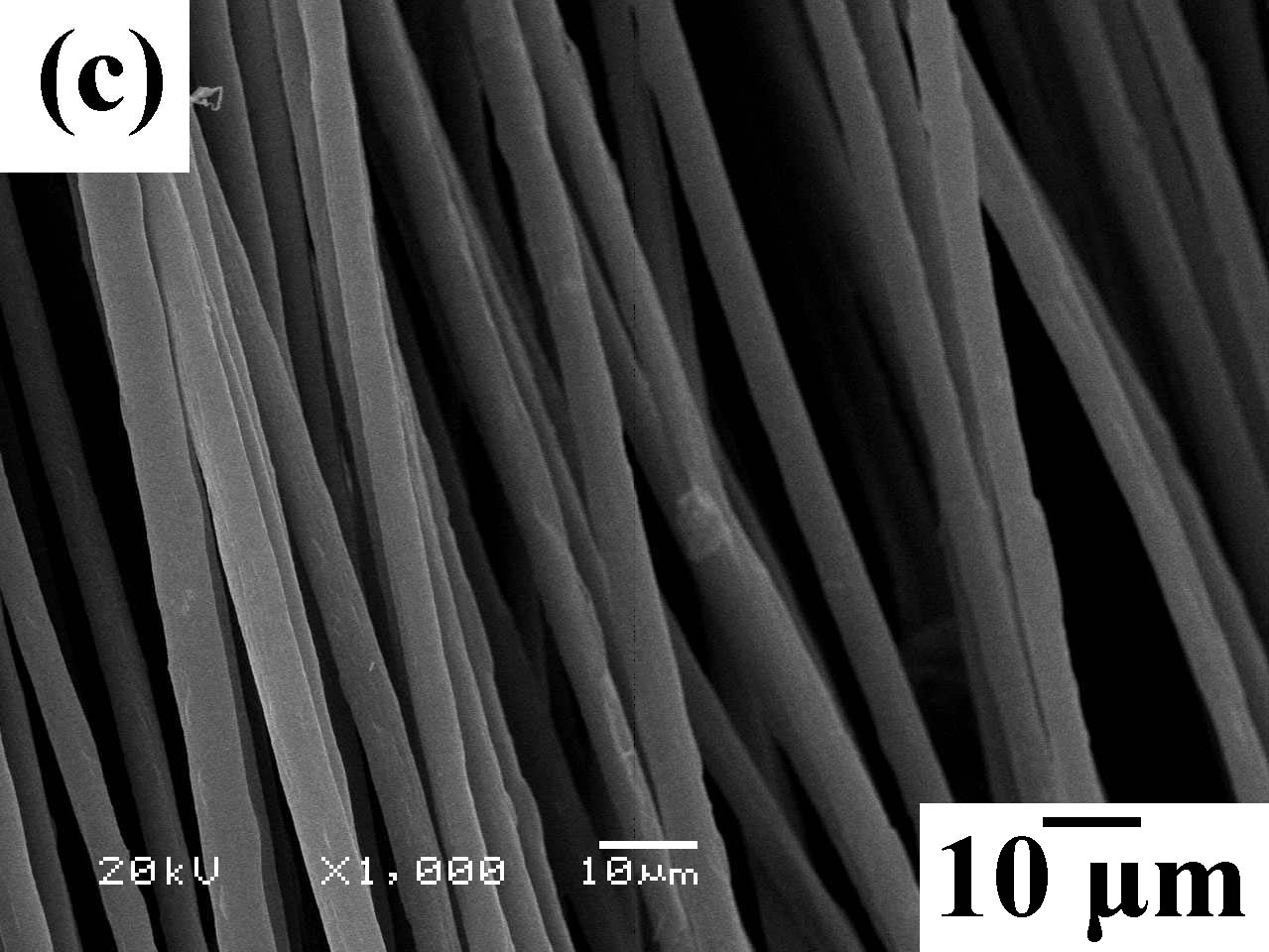


**Supplementary Figure.4** SEM images of the precursor TiO_2_ fibres obtained from different concentrations of Ti(OC_4_H_9_)_4_): (a) 8.0 wt%; (b) 10.0 wt %; (c) 20.0 wt %. The concentration of PEO was fixed to 2.0 wt %; the rate of rotation of the collecting drum was 1400 rpm. Here, 1 rpm was equivalent to a linear velocity of 0.0067 m/s.


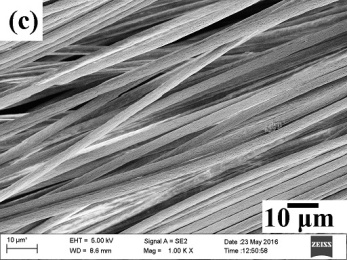

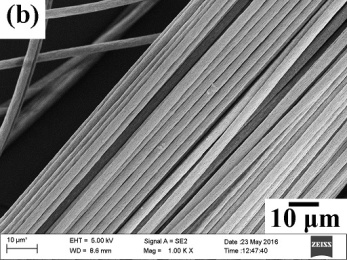

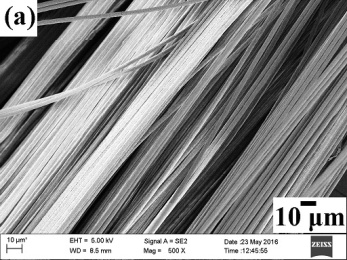


**Supplementary Figure.5** SEM images of the precursor TiO_2_ fibres obtained from different applied voltages: (a) 5.0 kV; (b) 8.0 kV; (c) 10.0 kV. The concentration of PEO was fixed at 2.0 wt %; the rate of rotation of the collecting drum was 1200 rpm. Here, 1 rpm was equivalent to a linear velocity of 0.0067 m/s.


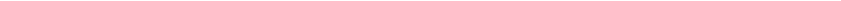


**Supplementary Figure.6** (a) XRD patterns of well-aligned TiO_2_:Eu fibre arrays with different Eu^3+^ dopants calcined at 500 °C; (b) XRD patterns of well-aligned TiO_2_:Sm fibre arrays with different Sm^3+^ dopants calcined at 600 °C; (c) XRD patterns of well-aligned TiO_2_:Eu fibre arrays calcined at different temperatures; (d) XRD patterns of well-aligned TiO_2_:Sm fibre arrays calcined at different temperatures. (e) XRD pattern of well-aligned TiO_2_:Er fibre arrays calcined at 600 °C.


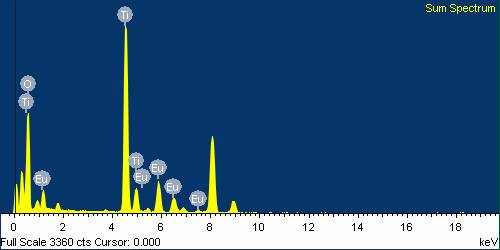


**Supplementary Figure.7.** EDX of the TiO_2_ fibre. It can be easily found that there exist the signal of Ti, O, Eu and Cu. The signal of Cu at 8.0 keV belongs to the conductive plating.


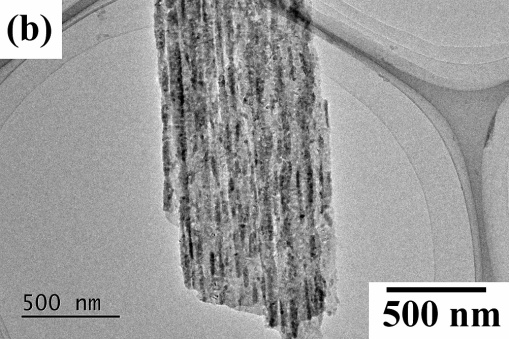

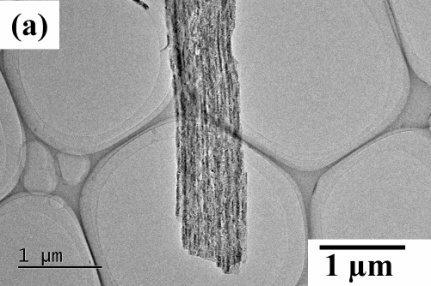

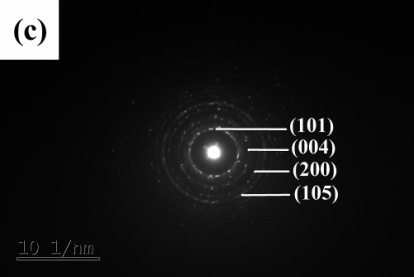


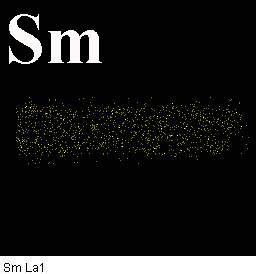

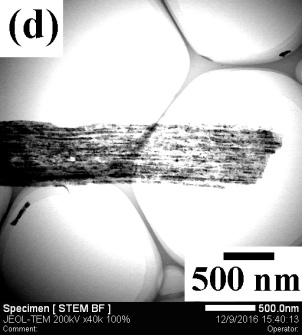

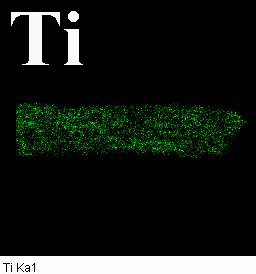

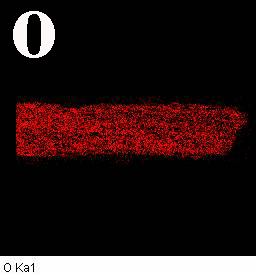

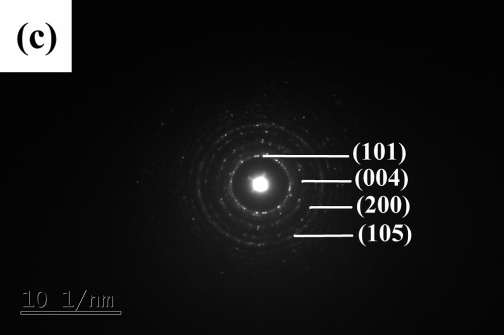


**Supplementary Figure.8.** (a) and (b) are lower magnification, image of the end, and HR-TEM images of a single pure TiO_2_:Sm fibre; (c) is an SAED pattern of the single pure TiO_2_:1.0 mol % Sm fibre. (d) STEM images and EDX elemental mapping of Ti, O and Sm of TiO_2_: Sm fibre. The as-prepared well-aligned fibres were taken off and calcined at a heating rate of 10 °C·h^-1^ and kept at 500 °C in air for 2 h.


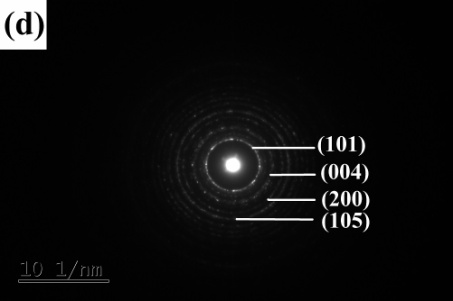

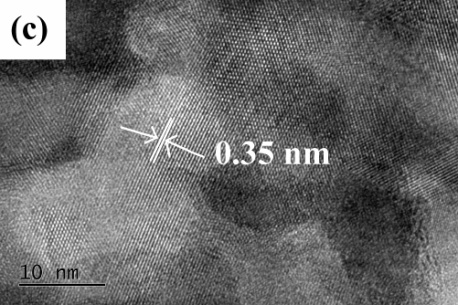

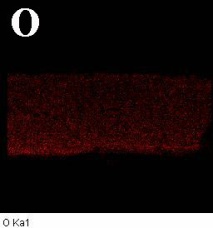

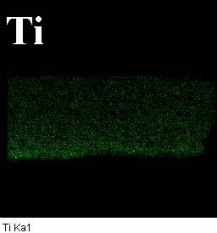

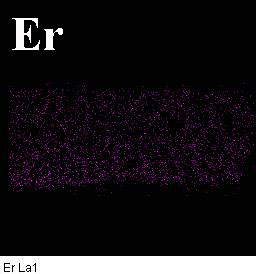

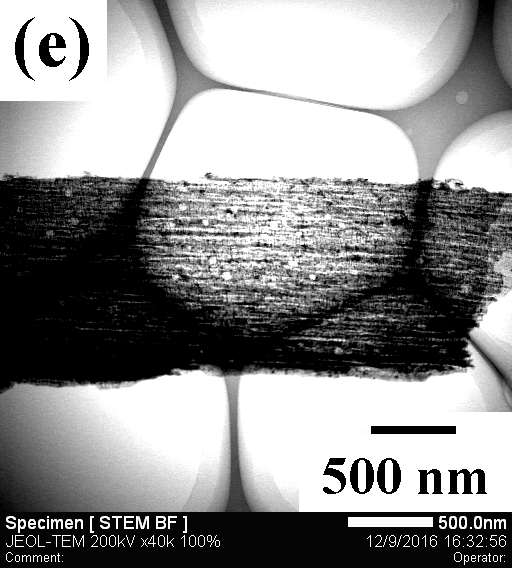

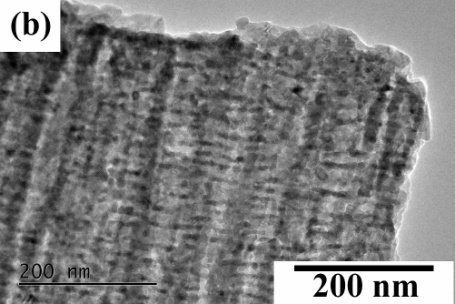

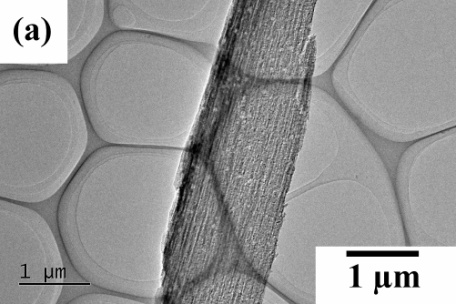


**Supplementary Figure.9.** (a) and (b) are lower magnification, image of the end, and HR-TEM images of a single pure TiO_2_:Er fibre; (c) is an SAED pattern of the single pure TiO_2_:1.0 mol % Er fibre. (d) STEM images and EDX elemental mapping of Ti, O and Sm of TiO_2_: Er fibre. The as-prepared well-aligned fibres were taken off and calcined at a heating rate of 10 °C·h^-1^ and kept at 500 °C in air for 2 h.


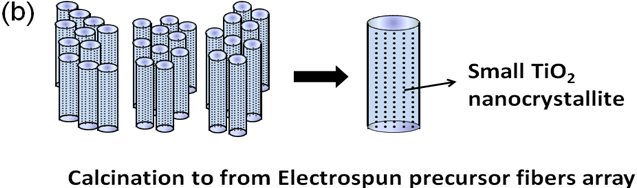

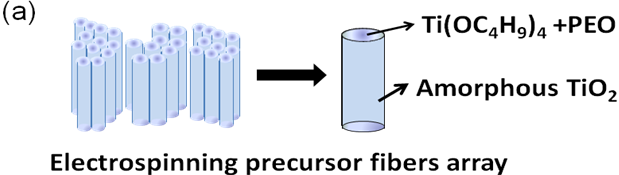


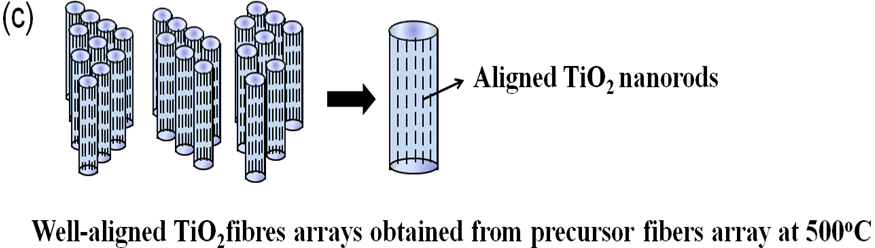


**Supplementary Figure.10.** Schematic diagram of the formation mechanism of well-aligned electrospun TiO_2_ fibre arrays.
